# A cross-sectional study on the application of patient-reported outcome measurements in clinical trials of traditional Chinese medicine in mainland China

**DOI:** 10.3389/fphar.2023.1159906

**Published:** 2023-05-11

**Authors:** Yue Dong, Lin Liu, Xiaowen Zhang, Yijia Gong, Shiyan Yan, Wei Li, Shunping Li, Hongguo Rong, Jianping Liu

**Affiliations:** ^1^ School of Traditional Chinese Medicine, Beijing University of Chinese Medicine, Beijing, China; ^2^ Center for Evidence-Based Chinese Medicine, Beijing University of Chinese Medicine, Beijing, China; ^3^ Institute for Excellence in Evidence-Based Chinese Medicine, Beijing University of Chinese Medicine, Beijing, China; ^4^ College of Acupuncture and Massage, Beijing University of Chinese Medicine, Beijing, China; ^5^ International Research Center for Medicinal Administration, Peking University, Beijing, China; ^6^ Centre for Health Management and Policy Research, School of Public Health, Cheeloo College of Medicine, Shandong University, Jinan, China

**Keywords:** traditional Chinese medicine, clinical trial, patient-reported outcomes, secondary outcomes, primary outcomes

## Abstract

**Objectives:** Patient-reported outcomes (PROs) provide a global perspective of patient health status which plays an enormous role in evaluating clinical efficacy. However, the application of PROs in traditional Chinese medicine (TCM) was still insufficiently studied in mainland China.

**Methods:** This cross-sectional study was performed based on interventional clinical trials of TCM that were conducted in mainland China from 1 January 2010, to 15 July 2022. Data was retrieved from the ClinicalTrials.gov and Chinese Clinical Trial Registry. We included interventional clinical trials of TCM for which the country of the primary sponsors or recruitment settings in mainland China. For each included trial, data including clinical trial phases, study settings, participant’s age, sex, diseases, and the patient-reported outcome measures (PROMs) were extracted. Trials were categorized into four categories according to 1) listed PROs as primary endpoints, 2) listed PROs as secondary endpoints, 3) listed PROs as coprimary outcomes (both primary and secondary endpoints), and 4) did not mention any PROMs.

**Results:** Among a total of 3,797 trials, 680 (17.9%) trials listed PROs as primary endpoints, 692 (18.2%) trials listed PROs as secondary endpoints, and 760 (20.0%) trials listed PROs as coprimary endpoints. Among 675,787 participants included in the registered trials, 448,359 (66.3%) patients’ data were scientifically collected by PRO instruments. Neurological diseases (11.8%), musculoskeletal symptoms (11.5%), mental health conditions (9.1%) were the most common conditions evaluated by PROMs. Disease-specific symptoms related concepts were used most frequently (51.3%), followed by health-related quality of life concepts. Visual analog scale, 36-item Short-Form Health Questionnaire, and TCM symptom score were the most common PROMs in these trials.

**Conclusion:** In this cross-sectional study, the use of PROs increased in the past decades according to clinical trials of TCM conducted in mainland China. Considering that the application of PROs in clinical trials of TCM has some existing issues including uneven distribution and lack of normalized PROs of TCM, further study should be focused on the standardization and normalization of TCM-specific scales.

## Introduction

Patient-reported outcomes (PROs) are any report of the status of a patient’s health condition that directly comes from the patient, without interpretation of the patient’s response by a clinician or anyone else (Patrick et al., 2007). Patient-reported outcome measures (PROMs) were the standardized questionnaires that collect information on health outcomes directly from patients (Churruca et al., 2021). PROs and PROMs were widely used in evaluating health-related quality of life, physical capacity, mental and cognitive changes, functional status, symptoms, and overall wellbeing (Casey, 2022). Over the past decades, healthcare systems have increasingly perceived patients' opinions as the fundamental condition to ensure that a high-quality, equitable, and safe service was delivered (Marshall et al., 2006). PROs provide evidence for supporting clinical decision making, prioritizing the surgical procedures of the patients, comparing outcomes among healthcare providers, promoting quality of treatment, and evaluating practices and policies (Dawson et al., 2010; Black, 2013; Chen et al., 2013; Chan et al., 2016; Price et al., 2019).

Traditional Chinese medicine (TCM) originated in mainland China with a history of more than 2,000 years and has been widely used in clinical practice. TCM plays an important role in global major public health emergencies, especially during the COVID-19 pandemic (Dai et al., 2021). The development of TCM Basic Quality of Life (ChQOL) demonstrates an evidence-based approach that can evaluate the therapeutic effectiveness of TCM through PROs or health-related quality of life measurements. With PROs becoming increasingly important in medicine, an increasing number of clinical trials of TCM used PROs as primary or secondary outcomes (Kochar et al., 2018; Santosa et al., 2018; Kotecha et al., 2020; Jia et al., 2021). TCM practitioners intend to focus on the patient as a unified person instead of individual symptoms, as Western medicine does (Zhang and Chor, 2023). PROs share a similar concept with the TCM approach (Jiang et al., 2010), which often represents the effect of diseases on all aspects of health and functioning (U.S. Department of Health and Human Services FDA Center for Drug Evaluation and Research U.S. Department of Health and Human Services FDA Center for Biologics Evaluation and Research U.S. Department of Health and Human Services FDA Center for Devices and Radiological Health, 2006). Using PROs, which are accepted by Western doctors, to evaluate the TCM clinical effect could form the basis for further testing and applications of TCM in Western countries (Leung et al., 2005; Zhao and Chan, 2005). It is widely believed among TCM practitioners that the current quality of life instruments may not be sufficiently sensitive to detect the changes in body status/symptoms that are deemed crucial in TCM treatment (Jiang et al., 2010).The patients' input and subjective experience were important elements to assess TCM treatment outcomes, which was consistent with the core concept of PROs (Zhang et al., 2017). Over the past several years, PROs have come into widespread use in the world, including China. However, China still faces many challenges in the development and implementation of PROs involving the large population, socio-economic status, geographical location, healthcare labor supply and many other variables. A cross-sectional study of clinical trials conducted in China indicated that only 29.7% of the selected eligible trials including PROs precisely listed PROMs as outcomes (Zhou et al., 2022). Meanwhile, lack of comprehensive evaluations assessing the application of PROs in clinical trials of TCM in mainland China.

Over the past several years, the number of clinical trials of TCM conducted in mainland China increased consistently. It is necessary to investigate the application of PROs in clinical trials of TCM to understand how PROs were being used and provide suggestions for conducting high-quality clinical trials of TCM. Based on the registration information of randomized clinical trials of TCM conducted in China, this study was aimed at reviewing and evaluating the use of PROs and providing potential study directions for future clinical practice of TCM.

## Methods

### Study design

This cross-sectional study analyzed the data from clinical trials of TCM conducted in mainland China with PROMs to evaluate primary and/or secondary outcomes from 1 January 2010 to 15 July 2022. Data was collected from ClinicalTrials.gov and Chinese Clinical Trial Registry. Only intervention studies in the two databases were retrieved (search strategy in the Supplementary Method S1). In light of these data, this study focused on these PROMs which were continually utilized in the conditions. The Strengthening the Reporting of Observational Studies in Epidemiology (STROBE) reporting guideline was followed in this study.

### Data collection strategy

**FIGURE 1:**
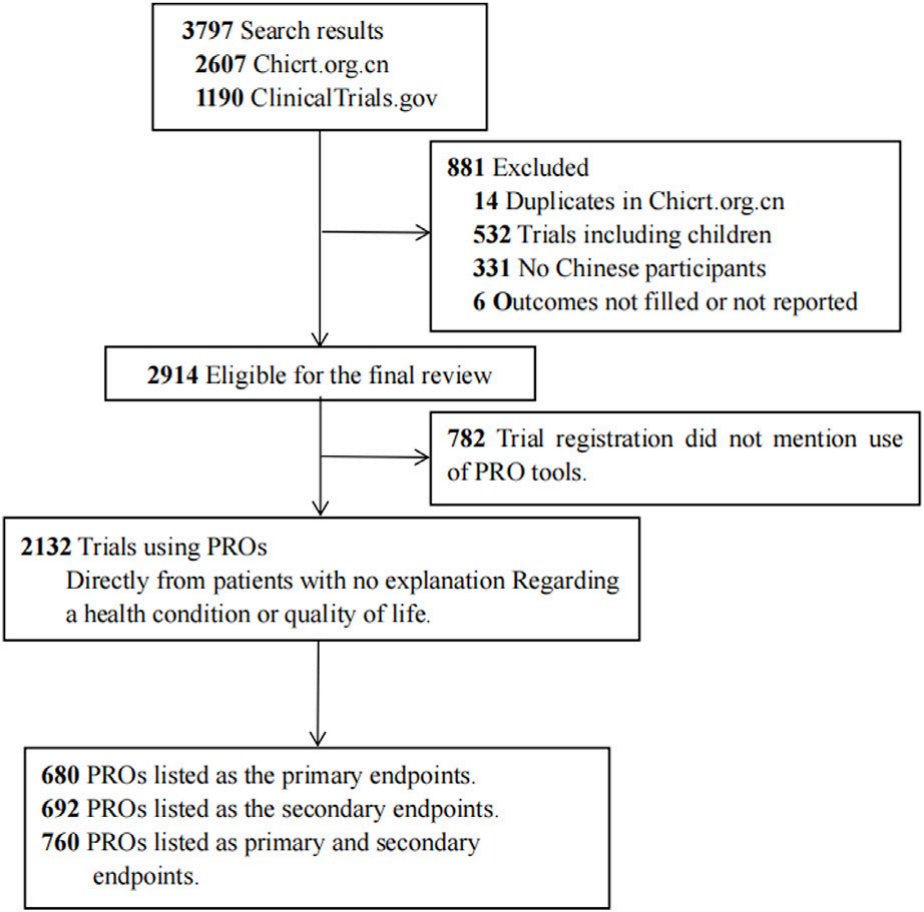
Trial exclusion and classification criteria.

We included interventional randomized clinical trials of TCM where the country of the primary sponsors or recruitment settings in mainland China, and recruited participants who were older than 18 years (Figure 1). In cases where the age of participants was “unclear,” we determine whether the trial involves children based on a comprehensive evaluation of the trial’s overall characteristics, such as the trial introduction, target diseases, and other relevant details. We excluded trials with duplicate registration numbers (retain ClinicalTrials.gov). Information collected to assess the conditions and characteristics of trials included 1) basic information, such as registration number, date of registration, official title, country, and uniform resource locator (URL), 2) key information, such as outcomes (including PROs), target disease, participant age and gender, inventions, and 3) feature information, such as primary sponsor, primary sponsor’s address, countries of recruitment, study settings, and clinical trial phases.

### Data classification

Eligible trials were classified into four categories according to the outcomes reported: 1) trial registration listed PROs as primary endpoints, 2) listed PROs as secondary outcome. 3) listed PROs as coprimary outcome (both primary and secondary endpoints), and 4) did not mention any PROMs (trial registration did not mention the use of PROs).

### Statistical analysis

The data from included trials were extracted independently by two authors (D.Y. and L.L.), using predesigned data extraction tables. Among them, the clinical trial phase, study setting, participant age and gender, region of the primary sponsor, center, and interventions of TCM were shown in Table 1. Owing to the varied categories and wide variation of target diseases, we classified similar target diseases as the same groups according to the International Classification of Diseases-11 (Supplementary Table S1 in the Supplement). Based on our categorization of conditions, our study summarized the PRO instruments used in each trial to calculate the most used measurements. We include only items that list the names of PRO tools in statistical analysis for quantitative analysis to understand which evaluation tools were used. Descriptive statistics were performed with Stata version 14.0 (StataCorp).

**TABLE 1:** Characteristics of all trials and trials of TCM including PROs.

		Total, No. (%)	
Characteristics		Trials	Trials using PROs
No.		2,914	2,132
Clinical trial phases			
	Early stage	846 (29.0)	615 (28.8)
	1	159 (5.4)	113 (5.3)
	2	139 (4.7)	108 (5.0)
	3	65 (2.2)	52 (2.4)
	4	366 (12.5)	249 (11.6)
	Other^a^	508 (17.4)	373 (17.4)
	Unclear	831 (28.5)	622 (29.1)
Study settings			
	Hospital	2,606 (89.4)	1,913 (89.7)
	Community	22 (0.7)	14 (0.6)
	Other^b^	286 (9.8)	205 (9.6)
Age			
	18-no limit	2,511 (86.1)	1,830 (85.8)
	Over 65	2 (0.1)	1 (0.1)
	Unclear	401 (13.7)	301 (14.1)
Gender			
	Male	117 (4.0)	83 (3.8)
	Female	366 (12.5)	249 (11.6)
	Both	2,426 (83.2)	1,796 (84.2)
Regions, mainland China			
	Southwest	294 (10.0)	219 (10.2)
	Northeast	85 (2.9)	64 (3.0)
	North	852 (29.2)	633 (29.6)
	Northwest	67 (2.2)	41 (1.9)
	East	1,078 (36.9)	780 (36.5)
	South	388 (13.3)	287 (13.4)
	Central	144 (4.9)	106 (4.9)
	Other^c^	6 (0.2)	2 (0.1)
No. of test centers			
	Single-center	2,276 (78.1)	1,673 (78.4)
	Multi-center	618 (21.2)	446 (20.9)
	Unclear	20 (0.6)	13 (0.6)
Chinese medicine interventions			
	Chinese herbal medicines	1,288 (44.2)	854 (39.7)
	Acupuncture	962 (33.0)	742 (35.3)
	Massage (Tui na)	82 (2.8)	66 (3.0)
	Cupping (Ba guan)	10 (0.4)	9 (0.4)
	Other^d^	572 (19.5)	461 (21.9)

^a^ Investigator-initiated trials, new treatment measurements, inspection technology, health service, and therapeutic devices.

^b^ Rehabilitation, nursing home, campus, centers for disease control, home, and research institute.

^c^ The trials were conducted in mainland China, but their sponsor was overseas.

^d^ Exercises, medicinal food, 5-element music therapy, combination therapy using multiple interventions.

## Results

### Trial characteristics

The general characteristics of the included trials were presented in Table 1. We identified 3,797 interventional studies conducted in mainland China, including 2,607 from the Chicrt.org.cn and 1,190 from ClinicalTrials.gov. Our study excluded 883 trials, including 14 duplicates, 532 clinical trials with participants younger than 18 years old, 331 trials not conducted in mainland China, and 6 trials with incomplete reporting - whose outcome measures/outcomes were not posted on ClinicalTrials.gov or the Chinese registries (Figure 1). Among the 3,797 included trials, 2,132 (56.1%) trials used PRO instruments as their primary and/or secondary endpoints, 782 (20.6%) trials did not mention the use of PROMs.

**FIGURE 2:**
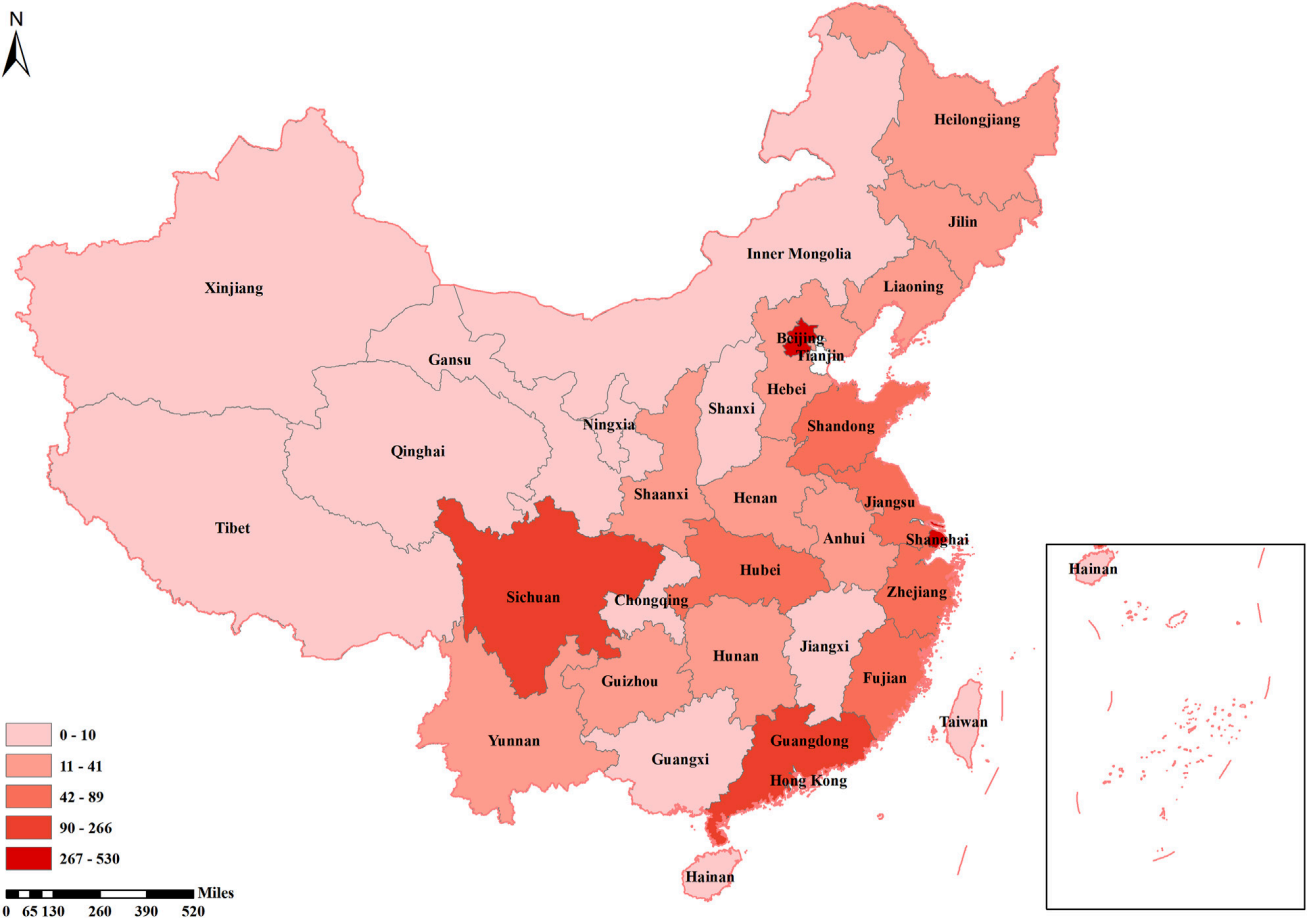
The number of trials with Patient-Reported Outcomes in each province.

Of the all clinical trials, early-stage trials [846/2914 (29.0%)] were the most common, followed by phase-4 (12.5%). Of the 2132 trials including PROs, early-stage trials [615/2132 (28.8%)] were still the most common, same followed by phase 4 (11.6%) trials (Table 1). Nearly 90% [2606/2914 (89.4%)] of the trials were conducted in hospitals, and less than 1% (0.7%) were performed in the community. Most primary sponsors were seated in eastern mainland China, followed by northern, southern, and southwestern mainland China; the other regions, including central, northeastern and northwestern mainland China, accounted for 1%–5%. There were similar findings considering only trials including PROs, with nearly 90% [1919/2,132 (90.0%)] of primary sponsors coming from the eastern, northern, southern, and southwest areas of mainland China; less than 10% of primary sponsors were from the central, northeastern, and northwestern mainland China (Table 1). There were considerable differences in the proportion of primary sponsors of trials including PROs among the different provinces. The percentage of primary sponsors of trials including PROs in Chinese provinces is shown in Figure 2.

We counted the number of implementation centers of trials included. 78.1% (*n* = 2,276) trials were performed at single center, and 21.2% (*n* = 618) trials were conducted at multiple centers. For interventions of TCM used in clinical trials, 40% (1,288/72,914) trials used Chinese herbal medicines as interventions, followed by acupuncture (33.0%), and exercises (11.2%). There were similar findings when considering only the trials including PROs.

### Health conditions and PROs

**FIGURE 3:**
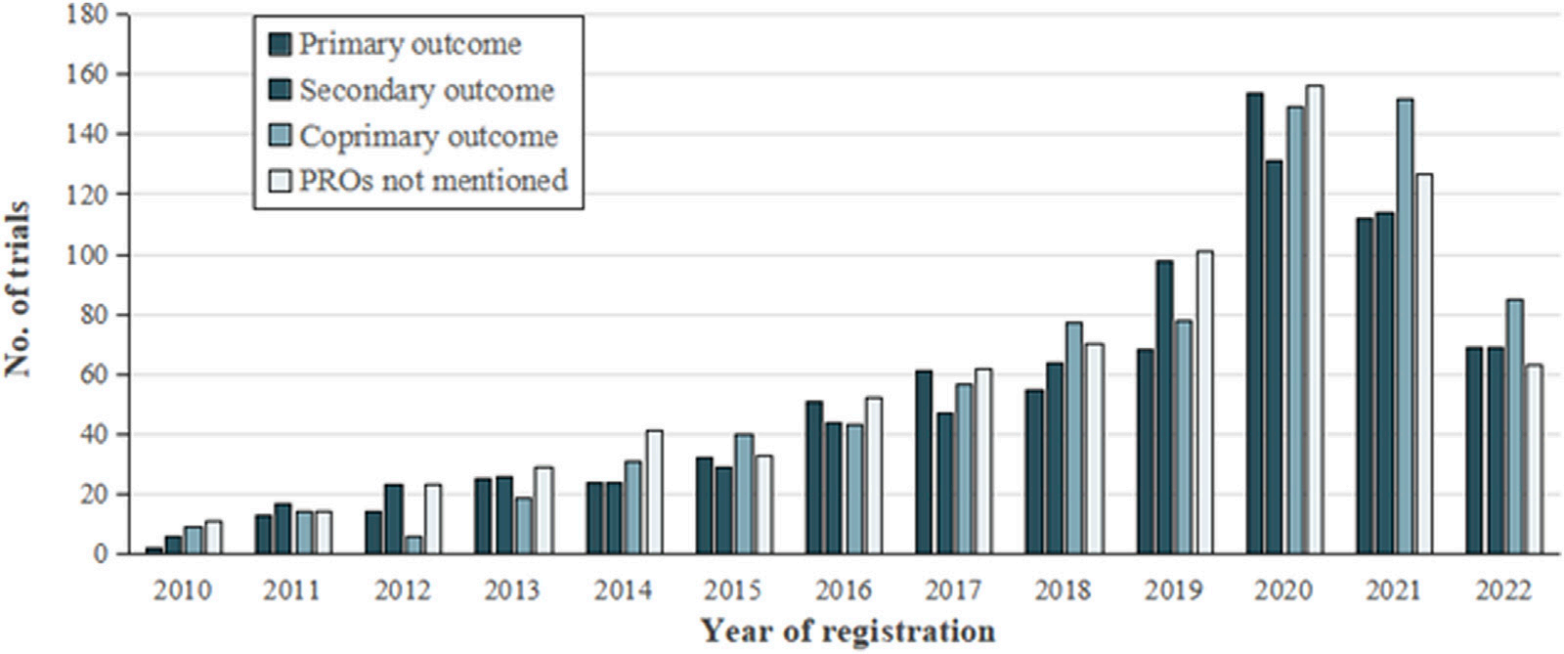
Number of clinical trials analyzed.

**FIGURE 4:**
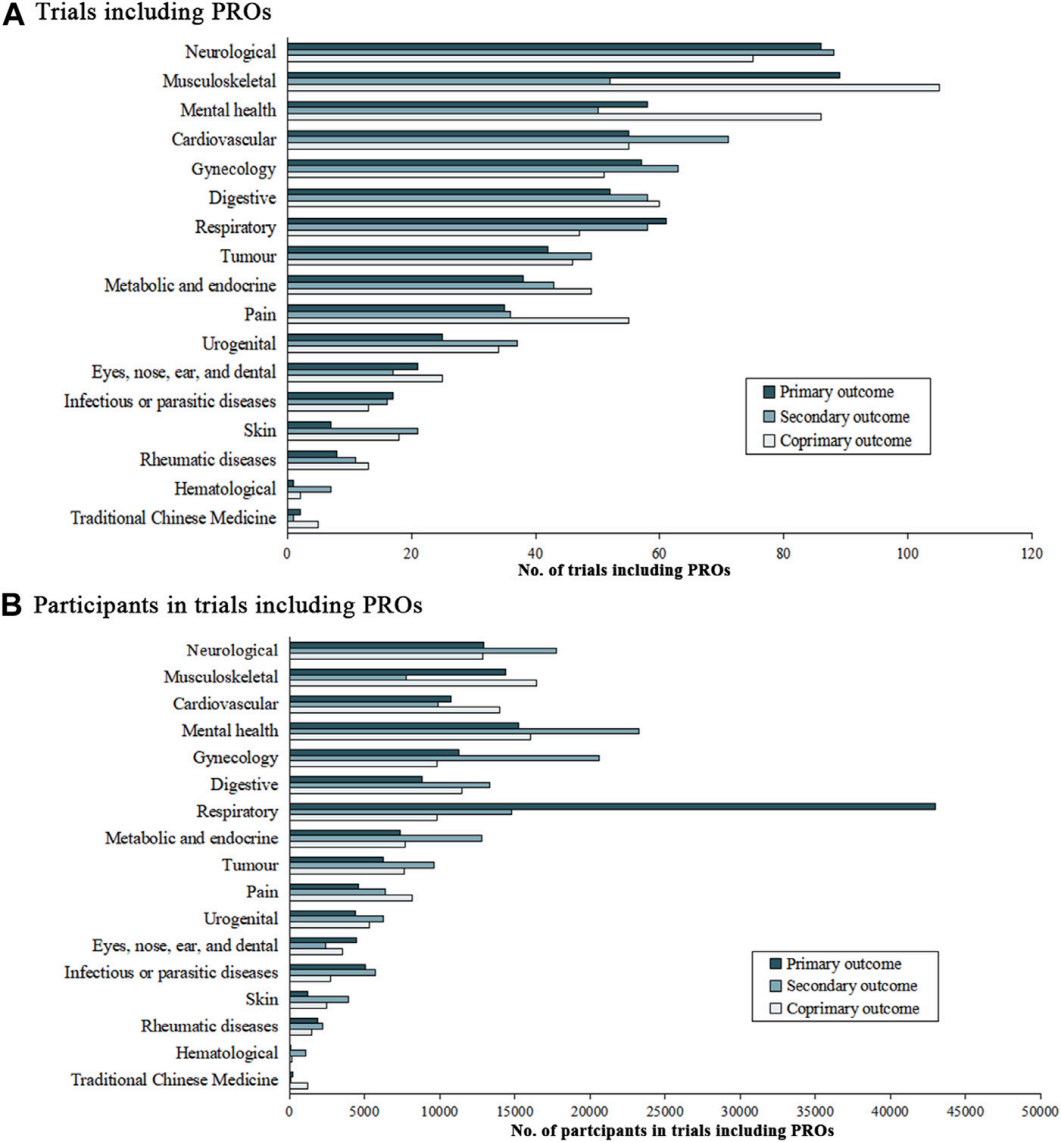
Number of trials and participants with patient-reported outcomes. No. of trials including PROs for **(A)**, and No. of participants in trials including PROs for **(B)**.

Figure 3 illustrates the increase in the number of TCM clinical registration trials from 2010 to 2022, and demonstrates the proportion of trials that list PRO as the outcome among the TCM trials. In the 2,132 trials that used PRO instruments, neurological (11.8%), musculoskeletal (11.5%), mental health conditions (9.1%), cardiovascular (8.5%), and gynecology diseases (8.0%) were the top five conditions which applied PRO measures as outcomes (Figure 4). Although the instruments used were varied by disease type, we found the most frequently used PROMs in these trials were visual analog scale (VAS), 36-item Short-Form Health Questionnaire (SF-36), and TCMSS (Supplementary Table S2 in the Supplement).

The number of conditions and participants in trials that included PROMs were shown in Figure 4. In the 1,440 trials that listed PROs as the primary endpoints, musculoskeletal diseases (13.5%), neurological diseases (11.2%), and mental health condition (10.0%) were considered as the most common conditions, followed by cardiovascular symptoms (7.6%), digestive (7.8%), respiratory (7.5%), and gynecology (7.5%) conditions. Pain, tumor, metabolic and endocrine, urogenital and eyes, nose, ear, and dental conditions accounted for 3%–7% of these trials. Infectious or parasitic disease conditions (2.1%), skin diseases (1.7%), rheumatic diseases (1.5%), TCM symptoms (0.5%), and hematological (0.2%) were the least common conditions considered.

Of the 448,359 individuals in trials that included PROs data, 15.1% (*n* = 67,605) were diagnosed with respiratory diseases, 12.2% (*n* = 54,499) were experiencing mental health condition and 9.7% (*n* = 43,471) had neurological conditions. Participants with gynecology [41,616 (9.3%)], musculoskeletal [38,576 (8.6%)], cardiovascular [34,637 (7.7%)], digestive [33,565 (7.5%)], metabolic and endocrine [27,790 (6.2%)] and tumor [23,392 (5.2%)] conditions were also well represented (>20,000 participants in all cases). More than 10,000 participants in these trials had pain [19,081 (4.3%)], urogenital [15,959 (3.6%)], infectious or parasitic diseases [13,474 (3.0%)] and eyes, nose, ears, and dental [10,294 (2.3%)] conditions. Less than 10,000 participants in this group had skin diseases [7,502 (1.7%)], rheumatic diseases [5575 (1.2%)], traditional Chinese medicine symptoms [1,522 (0.3%)], and hematological diseases [1,288 (0.3%)].

Consistent with previous systematic reviews in this setting (Gnanasakthy et al., 2022), trial outcomes were classified into four categories: symptoms, function, health-related quality of life (HRQOL), and others. Outcomes related to the kind of clinical manifestations (e.g., hot flash, pain, and bellyache) were classified as symptoms; the category function included concepts such as physical functioning, activity limitation, and emotional function were classified as function; changes in a patient’s quality of life, HRQOL, and perceived wellbeing were classified as HRQOL; outcomes related to patients’ satisfaction with treatment or feasibility were classified as other. Pre-specified concepts of PRO-related outcomes were italicized.

Table 2 shows the classification based on the scale content. Symptoms were reported in 1,947 trials, of which 596 were primary outcomes, 591 were secondary outcomes and 760 were coprimary outcomes. 582 reported trials focused predominantly on function. Function was used to support primary outcomes in 169 trials, secondary outcomes in 126 trials and coprimary outcomes in 287 trials. Of the 942 HRQOL trials, 425 (24.9%) were based on coprimary outcomes related to HRQOL.

**TABLE 2:** Classification according to the content of the patient-reported outcomes evaluation.

	PRO instruments conditions				
Trials conditions	Proportion	Symptoms	Function	HRQOL	Other
	No. (%)				
Total no.	2,132				
Primary	680 (31.9)	596/680 (87.6)	169/680 (24.9)	185/680 (27.2)	24/680 (3.5)
Secondary	692 (32.5)	591/692 (85.4)	126/692 (18.2)	332/692 (48.0)	81/692 (11.7)
Coprimary	760 (35.6)	760/692 (93.3)	287/692 (37.8)	425/692 (55.9)	78/692 (10.3)

To determine the accurate application of different PROMs in trials including PROs, we categorized similar target diseases into 15 conditions according to ICD-11 (Tables 3–5). The VAS was used in 17.4% of neurological condition trials and 31.5% of musculoskeletal condition trials to assess primary outcomes. Of 181 identified trials with cardiovascular disease trials and 191 gynecology condition trials, TCMSS was the most-used PROMs.

**TABLE 3:** Frequency of the use of PROMs as primary outcome in different classification of trials of TCM by condition.

		PRO instruments						
Conditions	Proportion No. (%)	No./total no. (%)	Name	No./total no. (%)	Name	No./total no. (%)	Name	No./total no. (%)
Total no.	2,132							
Neurological	249 (11.7)	88/249 (35.3)	Traditional Chinese symptom score	11/88 (12.5)	SF-36	9/88 (10.2)	NIHSS	9/88(10.2)
Musculoskeletal	246 (11.5)	52/246 (21.1)	VAS	8/52 (15.4)	SF-36	5/52 (9.6)	TCMSS	5/52 (9.6)
Mental health	194 (9.1)	50/194 (25.8)	PSQI	6/50 (12.0)	TCMSS	5/50 (10.0)	TCM Symptom Rating Scale	5/50 (10.0)
Cardiovascular	181 (8.5)	71/181 (39.2)	SAQ	13/71 (18.3)	TCMSS	10/71 (14.1)	SF-36	9/71 (12.7)
Gynecology	171 (8.0)	63/171 (36.8)	SAS	8/63 (12.7)	TCMSS	7/63 (11.1)	VAS	7/63 (11.1)
Digestive	170 (8.0)	58/170 (34.1)	SF-36	7/58 (12.1)	VAS	7/58 (12.1)	SAS	5/58 (8.6)
Respiratory	166 (7.8)	58/166 (34.9)	VAS	9/58 (15.5)	SF-36	8/58 (13.8)	CAT	6/58 (10.3)
Tumour	137 (6.4)	49/137 (35.8)	EORTC QLQ-C30	4/49 (8.2)	SF-36	4/49 (8.2)	EQ-5D	3/49 (6.1)
Metabolic and endocrine	130 (6.1)	43/130 (33.1)	SF-36	5/43 (11.6)	TCMSS	5/43 (11/6)	VAS	5/43 (11.6)
Pain	126 (5.9)	36/126 (28.6)	VAS	9/36 (25.0)	TCMSS	7/36 (19.4)	HAMA	4/36 (11.1)
Urogenital	96 (4.5)	37/96 (38.5)	ICIQ-SF	7/37 (18.9)	TCMSS	5/37 (13.5)	TCM Symptom Rating Scale	4/37 (10.8)
Eyes, nose, ear, and dental	63 (3.0)	17/63 (27.0)	TCMSS	5/17 (29.4)	SAS	2/17 (7.4)	NRS	2/17 (7.4)
Skin	46 (2.2)	21/46 (45.7)	VAS	8/21 (38.1)	DLQI	7/21 (33.3)	Body surface area (BSA)	4/21 (19.0)
Infectious or parasitic diseases	46 (2.2)	16/46 (34.8)	TCMSS	3/16 (18.8)	FAQ	2/16 (12.5)	MOCA	2/16 (12.5)
Rheumatic diseases	32 (1.5)	11/32 (34.4)	TCMSS	2/11 (18.2)	DAS-28	1/11 (9.1)	QLQ-LC13	1/11 (9.1)

Abbreviations: CAT, COPD Assessment Test; DAS‐28, Disease Activity Score‐28; DLQI, Dermatology Life Quality Index; FAI, Fatigue rating scale; HAMA, Hamilton Anxiety Rating Scale; HAMD, Hamilton Depression Scale; IBS‐SSS, Irritable Bowel Syndrome Symptom Severity; ICIQ‐SF, International Consultation on Incontinence Questionnaire–Short Form; IPSS, International Prostate Symptom Score; IWQOL, Impact Weight Quality Of Life; JOA, Japanese Orthopaedic Association; MLHFQ, The Minnesota living with heart failure questionnaire; mMRC, Modified Medical Research Council scale; MMSE, Mini-mental State Examination; MoCA, Montreal cognitive assessment; MPQ, McGill Pain Questionnaire; MRS, Greene Climacteric Scale; MSQ, Migraine‐Specific Quality‐of‐Life Questionnaire; NDI, Neck Disability Index; NRS, numeric rating scale; OABSS, Overactive Bladder Symptom Score; ODI, Oswestry Disability Index; OSDI, Ocular Surface Disease Index; PDQ, Parkinson Disease Questionnaire; PFS, Piper Fatigue Survey Scale; PRO, patient-reported outcome; PSQI, Pittsburgh Sleep Quality Index; QLQ‐C30, Quality of Life Questionnaire–Core 30; SAQ, Seattle Angina Questionnaire; SAS, Self-rating Anxiety Scale; SDS, Self-rating Depression Scale; SF‐36, Short‐Form 36‐item Health Survey; SGRQ, St George's Respiratory Questionnaire; Skindex16, Skindex‐16 dermatologic survey; TCMSS, TCM symptom score; TFI, Tilburg Frailty Indicator; THI, Tinnitus Handicap Inventory; TNSS, Total nasal symptom score; VAS, visual analog scale; WHOQOL‐HIV BREF, World Health Organization Quality of Life HIV instrument; WOMAC, The Western Ontario and McMaster Universities Osteoarthritis Index.

**TABLE 4:** Frequency of the use of PROMs as coprimary outcome in different classification of trials of TCM by condition.

		PRO instruments						
Conditions	Proportion No. (%)	No./total no. (%)	Name	No./total no. (%)	Name	No./total no. (%)	Name	No./total no. (%)
Total No.	2,132							
Neurological	249 (11.7)	75/249 (30.1)	MMSE	18/75 (24)	VAS	12/75 (16)	PSQI	10/75 (13.3)
Musculoskeletal	246 (11.5)	105/246 (42.7)	VAS	41/105 (39.0)	SF-36	22/105 (21.0)	WOMAC	21/105 (20.0)
Mental health	194 (9.1)	86/194 (44.3)	PSQI	24/86 (27.9)	VAS	18/86 (20.9)	SAS	16/86 (18.6)
Cardiovascular	181 (8.5)	55/181 (30.4)	PSQI	14/55 (25.5)	VAS	13/55 (23.6)	SF-36	11/55 (20.0)
Gynecology	171 (8.0)	51/171 (29.8)	VAS	19/51 (37.3)	PSQI	8/51 (15.7)	SF-36	7/51 (13.7)
Digestive	170 (8.0)	60/170 (35.3)	VAS	17/60 (28.3)	SF-36	11/60 (18.3)	SDS	10/60 (16.7)
Respiratory	166 (7.8)	47/166 (28.3)	VAS	17/47 (36.2)	SDS	10/47 (21.3)	SAS	9/47 (19.1)
Tumour	137 (6.4)	46/137 (33.6)	PSQI	10/46 (21.7)	VAS	10/46 (21.7)	SDS	5/46 (10.9)
Metabolic and endocrine	130 (6.1)	49/130 (37.7)	VAS	10/49 (20.4)	PSQI	9/49 (18.4)	SF-36	6/49 (12.2)
Pain	126 (5.9)	55/126 (43.7)	VAS	26/55 (47.3)	SF-36	15/55 (27.3)	NRS	5/55 (9.1)
Urogenital	96 (4.5)	34/96 (35.4)	PSQI	8/34 (23.5)	VAS	7/34 (20.6)	NIH-CPSI	7/34 (20.6)
Eyes, nose, ear, and dental	63 (3.0)	25/63 (39.7)	VAS	5/25 (20.0)	PSQI	5/25 (20.0)	SF-36	3/25 (12.0)
Skin	46 (2.2)	18/46 (39.1)	VAS	6/18 (33.3)	TCMSS	4/18 (22.2)	SF-36	3/18 (16.7)
Infectious or parasitic diseases	46 (2.2)	13/46 (28.3)	VAS	5/13 (38.5)	SF-12	3/13 (23.1)	TCMSS	3/13 (23.1)
Rheumatic diseases	32 (1.5)	13/32 (40.6)	SF-36	8/13 (61.5)	VAS	7/13 (53.8)	BASFI	3/13 (23.1)

Abbreviations: CAT, COPD Assessment Test; DAS‐28, Disease Activity Score‐28; DLQI, Dermatology Life Quality Index; FAI, Fatigue rating scale; HAMA, Hamilton Anxiety Rating Scale; HAMD, Hamilton Depression Scale; IBS-SSS, Irritable Bowel Syndrome Symptom Severity; ICIQ-SF, International Consultation on Incontinence Questionnaire–Short Form; IPSS, International Prostate Symptom Score; IWQOL, Impact Weight Quality Of Life; JOA, Japanese Orthopaedic Association; MLHFQ, The Minnesota living with heart failure questionnaire; mMRC, Modified Medical Research Council scale; MMSE, Mini‐mental State Examination; MoCA, Montreal cognitive assessment; MPQ, McGill Pain Questionnaire; MRS, Greene Climacteric Scale; MSQ, Migraine-Specific Quality‐of‐Life Questionnaire; NDI, Neck Disability Index; NRS, numeric rating scale; OABSS, Overactive Bladder Symptom Score; ODI, Oswestry Disability Index; OSDI, Ocular Surface Disease Index; PDQ, Parkinson Disease Questionnaire; PFS, Piper Fatigue Survey Scale; PRO, patient‐reported outcome; PSQI, Pittsburgh Sleep Quality Index; QLQ‐C30, Quality of Life Questionnaire–Core 30; SAQ, Seattle Angina Questionnaire; SAS, Self-rating Anxiety Scale; SDS, Self-rating Depression Scale; SF‐36, Short‐Form 36-item Health Survey; SGRQ, St George's Respiratory Questionnaire; Skindex16, Skindex‐16 dermatologic survey; TCMSS, TCM symptom score; TFI, Tilburg Frailty Indicator; THI, Tinnitus Handicap Inventory; TNSS, Total nasal symptom score; VAS, visual analog scale; WHOQOL‐HIV BREF, World Health Organization Quality of Life HIV instrument; WOMAC, The Western Ontario and McMaster Universities Osteoarthritis Index.

**TABLE 5:** Frequency of the use of PROMs as secondary outcome in different classification of trials of TCM by condition.

		PRO instruments						
Conditions	Proportion No. (%)	No./total no. (%)	Name	No./total no. (%)	Name	No./total no. (%)	Name	No./total no. (%)
Total No.	2,132							
Neurological	249 (11.7)	75/249 (30.1)	MMSE	18/75 (24)	VAS	12/75 (16)	PSQI	10/75 (13.3)
Musculoskeletal	246 (11.5)	105/246 (42.7)	VAS	41/105 (39.0)	SF-36	22/105 (21.0)	WOMAC	21/105 (20.0)
Mental health	194 (9.1)	86/194 (44.3)	PSQI	24/86 (27.9)	VAS	18/86 (20.9)	SAS	16/86 (18.6)
Cardiovascular	181 (8.5)	55/181 (30.4)	PSQI	14/55 (25.5)	VAS	13/55 (23.6)	SF-36	11/55 (20.0)
Gynecology	171 (8.0)	51/171 (29.8)	VAS	19/51 (37.3)	PSQI	8/51 (15.7)	SF-36	7/51 (13.7)
Digestive	170 (8.0)	60/170 (35.3)	VAS	17/60 (28.3)	SF-36	11/60 (18.3)	SDS	10/60 (16.7)
Respiratory	166 (7.8)	47/166 (28.3)	VAS	17/47 (36.2)	SDS	10/47 (21.3)	SAS	9/47 (19.1)
Tumour	137 (6.4)	46/137 (33.6)	PSQI	10/46 (21.7)	VAS	10/46 (21.7)	SDS	5/46 (10.9)
Metabolic and endocrine	130 (6.1)	49/130 (37.7)	VAS	10/49 (20.4)	PSQI	9/49 (18.4)	SF-36	6/49 (12.2)
Pain	126 (5.9)	55/126 (43.7)	VAS	26/55 (47.3)	SF-36	15/55 (27.3)	NRS	5/55 (9.1)
Urogenital	96 (4.5)	34/96 (35.4)	PSQI	8/34 (23.5)	VAS	7/34 (20.6)	NIH-CPSI	7/34 (20.6)
Eyes, nose, ear, and dental	63 (3.0)	25/63 (39.7)	VAS	5/25 (20.0)	PSQI	5/25 (20.0)	SF-36	3/25 (12.0)
Skin	46 (2.2)	18/46 (39.1)	VAS	6/18 (33.3)	TCMSS	4/18 (22.2)	SF-36	3/18 (16.7)
Infectious or parasitic diseases	46 (2.2)	13/46 (28.3)	VAS	5/13 (38.5)	SF-12	3/13 (23.1)	TCMSS	3/13 (23.1)
Rheumatic diseases	32 (1.5)	13/32 (40.6)	SF-36	8/13 (61.5)	VAS	7/13 (53.8)	BASFI	3/13 (23.1)

Abbreviations: CAT, COPD assessment test; DAS-28, disease activity score-28; DLQI, dermatology life quality index; FAI, fatigue rating scale; HAMA, hamilton anxiety rating scale; HAMD, hamilton depression scale; IBS-SSS, irritable bowel syndrome symptom severity; ICIQ-SF, international consultation on incontinence questionnaire–short form; IPSS, international prostate symptom score; IWQOL, impact weight quality of life; JOA, japanese orthopaedic association; MLHFQ, the minnesota living with heart failure questionnaire; mMRC, modified medical research council scale; MMSE, mini-mental state examination; MoCA, montreal cognitive assessment; MPQ, McGill pain questionnaire; MRS, greene climacteric scale; MSQ, migraine-specific quality-of-life questionnaire; NDI, neck disability index; NRS, numeric rating scale; OABSS, overactive bladder symptom score; ODI, oswestry disability index; OSDI, ocular surface disease index; PDQ, parkinson disease questionnaire; PFS, piper fatigue survey scale; PRO, patient-reported outcome; PSQI, pittsburgh sleep quality index; QLQ-C30, quality of life questionnaire–Core 30; SAQ, seattle angina questionnaire; SAS, self-rating anxiety scale; SDS, self-rating depression scale; SF-36, short-form 36-item health survey; SGRQ, St George’s respiratory questionnaire; Skindex16, Skindex-16, dermatologic survey; TCMSS, TCM symptom score; TFI, tilburg frailty indicator; THI, tinnitus handicap inventory; TNSS, total nasal symptom score; VAS, visual analog scale; WHOQOL-HIV BREF, world health organization quality of life HIV instrument; WOMAC, The western Ontario and McMaster universities osteoarthritis index.

Considering PROs were secondary outcomes, PROMs were used as follows. TCMSS and SF-36 were widely used in neurological conditions. Trials focused on urogenital conditions tended to use International Consultation on Incontinence Questionnaire-Short Form (ICIQ-SF) (18.9%) to assess urinary incontinence.

In trials reporting PROs as coprimary outcomes, Mini-mental State Examination (MMSE) was used as chief instrument to access the neurological condition. Besides, the preliminary data suggested trials for mental health, cardiovascular, tumor, and urogenital conditions had a tendency to use Pittsburgh Sleep Quality Index (PSQI) to evaluate their coprimary outcomes. The VAS was reported as coprimary outcome measure in all trials. However, SF-36 was frequently focused on in trials of musculoskeletal and digestive conditions. For the 2,315 trials included PROs, the VAS (25.7%), TCMSS (14.6%), SF-36 (11.8%), PSQI (11.1%), and Self-Rating Anxiety Scale (SAS) (9.4%) were the top five measurements used.

## Discussion

This cross-sectional study analyzed the application and characteristics of the PROs in randomized clinical trials of TCM conducted in mainland China from 2010 to 2022. Our study found that 20% of the eligible trials used PRO instruments as coprimary outcomes to assess the subjective perception of patients. We proposed that the other 80% of the trials neglected subjective evaluations of patients. Our results also indicated that the standard and widely used PRO instruments for TCM were not enough, which was inconsistent with our anticipation. Introducing PROs could benefit TCM assessment in clinical practice. It is significant to introduce PROs to TCM evaluation, which can improve the comprehensiveness, accuracy, and degree of individuation of the evaluation, while also contributing to the monitoring and adjustment of the treatment effect of TCM (Rubenstein et al., 1989; Roter, 2000; Gilbody et al., 2003; Cleland et al., 2009; Jiang et al., 2012; Calvert et al., 2018).

It is not surprising that neurological trials were the most common in clinical trials of TCM. Many TCM unique therapies have confirmed the beneficial effect on neurological diseases (Guo et al., 2014; Liu et al., 2019), and the patients' opinions were the key factor for the modernization of TCM (Lu et al., 2020). Meanwhile, the long-term consequences of the stroke event would be reduced during routine healthcare (Lynch et al., 2008). PROs could capture more subtle changes in the life of the stroke patient (Roberts and Counsell, 1998; Kaplan, 2003), which filled the gaps in the routine healthcare for the stroke patient.

Our study suggested that most PROMs were employed for evaluating the symptoms in clinical trials of TCM. TCM symptoms were basic units of TCM treatment, in addition to being key factors for the modernization of TCM (Lu et al., 2020). This should not be ignored that TCM has its unique advantages to treat complex diseases with holistic concept (Huang et al., 2020). To treat patients based on the holistic consideration of individuals’ health, more retinue health data are required. Therefore, PROs on function and HRQOL also deserve enough attention (Reich et al., 2012; Myles et al., 2017; Alghadir et al., 2018; Chiarotto et al., 2019).

Both the primary and secondary outcomes placed a strong emphasis on VAS, which was used to quickly and easily evaluate patients' status, especially symptoms. The reason for wide application of VAS could be that VAS assesses subjective indicators such as pain and discomfort (Kelly, 2001; Karcioglu et al., 2018), which were highly compatible with the assessment indicators commonly used in Western medical clinical research, facilitating the comparison and comprehensive analysis of research results. There was sufficient information on irregular and inadequate PROMs in the included trials. Our results suggested that the Traditional Chinese symptom score and TCM Symptom Rating Scale were widely used, but syndrome-specific scales, such as TCM-SDS, were still underutilized. Our study revealed that PROs in trials mainly concentrated on evaluating symptoms, functioning, and quality of life, while neglecting the assessment of patients' treatment expectations and satisfaction. We found some researchers had begun to independently design new PROMs, which were correctly targeted for TCM syndrome and more widespread in clinical trials (Zhang et al., 2012; Sun et al., 2021a; Jiang et al., 2021). The development of these scales suggested that efforts should be directed towards the development of standardized PROMs tailored to quantifying the various components of TCM consultations and enabling patients to evaluate their symptoms using a standardized methodology.

Early-stage clinical trials accounted for the largest proportion in all included trials, which suggested that patients’ participation was important for the evaluation of the effectiveness and safety of drugs (Haslam et al., 2020). The early stages of clinical trials were followed by Phase 4. Phase 4 clinical trials provided a better reflection of effectiveness, tolerability, and safety in the real world by evaluating TCM intervention among diverse, large and heterogeneous patient populations, thus informing regulatory and reimbursement decisions and contributing to health policy-making (Maruszczyk et al., 2022). The large percentage of Phase 4 indicated that patients’ subjective feelings should be more emphasized when the trial did not mainly concentrate on marketing.

In our study, the obvious regional difference in the use of PROs was found. PROs were more frequently adopted in eastern, northern, and southern mainland China, especially in Shanghai, Beijing, Sichuan, and Guangdong; while rarely in northwestern, and northeastern, especially in Qinghai and Tibet. This may be related to the level of political, economic, and medical development level of corresponding regions and provinces (Liniker et al., 2013). Remarkably, in trials conducted in remote regions such as Tibet and Qinghai, PROs may not be able to implement or need to be simplified, which may influence the selection of PRO instruments (Zhou et al., 2022).

PROs can provide a reference for the effectiveness and safety of interventions, while serving as the vital basis for labeling claims on noninnovative drug trials, such as bioequivalence studies (Dougados et al., 2013; Kyte et al., 2016; Rivera et al., 2019). Our study indicated that Chinese herbal medicine was the most commonly used in TCM intervention. This may be due to Chinese herbal medicine being licensed and widely recognized in mainland China, and they were therapeutic modalities that were frequently used in integrative medicine (Sun et al., 2021b; Weiwei, 2022). Previous studies suggested that Chinese herbal medicine has been used to treat a broad range of neurological conditions, including stroke, Alzheimer’s disease, and Parkinson’s disease (Yao et al., 2017).

The Chinese government released Guidelines for the Application of Patient-Reported Outcomes in Drug Clinical Research in 2021, and thought highly of the clinical advantages of PROs. However, the current PROs may lack the necessary sensitivity to capture essential information relevant to TCM (Jiang et al., 2010). Investigators should join their efforts in conducting high-quality trials to improve the well-established protocols of PRO in TCM trials and enrich PRO instruments based on the current status of trials of TCM including PROs. Given the unique and esoteric terminology used in TCM practices, such as “Shen Xu” and “Na Dai,” it is important to consider the promotion of PROMs that are accessible and comprehensible to individuals without a background in TCM education in clinical settings (Sun et al., 2021a).

## Limitation

Our study has several limitations. Firstly, considering young children may not express their feelings accurately, and parents reported outcomes could be influenced by multi-factors, we did not include studies of children to avoid the potential bias of the results. Secondly, only trials conducted in mainland China were included in our study. Thus, our results may be limited to generalize due to cultural and regional factors. Finally, during data extraction, we found that some registration information of trials was not updated. For instance, a number of trials that were registered a few years ago were still shown as being in the recruiting phase, which may cause bias in the sample size.

## Conclusion

In this cross-sectional study, the use of PROs increased in clinical trials of TCM conducted in mainland China in the past decades. Considering the application of PROs in clinical trials of TCM has some existing issues including uneven distribution and lack of standardized PROMs of TCM, further study should be focused on the development of standardized PROMs tailored to quantifying the various components of TCM consultations. It should be noticed that disease-specific versus generic scales for TCM trials need to be strongly developed.

## Data Availability

The original contributions presented in the study are included in the article/Supplementary Material, further inquiries can be directed to the corresponding author.
