# Supplementary material for: A cross-sectional study on the application of patient-reported outcome measurements in clinical trials of traditional Chinese medicine in mainland China
Source: Front Pharmacol. 2023 May 11;14:1159906. doi: 10.3389/fphar.2023.1159906 (PMC10213936; doi:10.3389/fphar.2023.1159906)
Supplement: Supplementary file 1 [file DataSheet1.PDF]

Supplement

e Methods. Search strategy

e Table 1. PRO tests used most frequently

e Table 2. Classification of specific diseases

e Methods. Search strategy

1. Search strategy for www.chictr.org.cn

"Study type" = Interventional Study

"Study phase" = 1, 2, 3, 4, 1-2, 2-3, 0, 4, N/A,

"First Posted" = "01/01/2010" To "15/07/2022"

"sex" = Male, Female, Both

"Country" = China

"Intervention/treatment"= TCM OR Chinese medicine OR traditional medicine OR ethnic medicine OR Chinese herbs OR materia medica OR herbal medicine OR decoction OR granules OR pills OR tablets OR powder OR capsule OR Chinese medicine injection OR patch OR acupuncture OR moxibu

Tai Chi OR Baduanjin OR Qi Gong OR Five Animal Play OR Yi Jin Jing OR massage OR Cupping OR Chinese and Western Medicine

2. Search strategy for ClinicalTrials.gov

"Study type" = Interventional Studies (Clinical Trials)

"Phase" = Early phase 1, Phase 2, Phase 3, Phase 4, Not Applicable

"First Posted" = "01/01/2010" To "31/12/2020"

"Sex" = Studies with Male Participants, Studies with Female Participants, All

"Country" = China

"Intervention/treatment"= TCM OR Chinese medicine OR traditional medicine OR ethnic medicine OR Chinese herbs OR materia medica OR herbal medicine OR decoction OR granules OR pills OR tablets OR powder OR capsule OR Chinese medicine injection OR patch OR acupuncture OR moxibu

Tai Chi OR Baduanjin OR Qi Gong OR Five Animal Play OR Yi Jin Jing OR massage OR Cupping OR Chinese and Western Medicine

e Table 1 Classification of specific diseases

1	Musculoskeletal	Knee Osteoarthritis	lumbar disc herniation	Cervical Spondylosis	Osteoarthritis
		Postmenopausal Osteoporosis	Osteoporosis	Cervical spondylopathy	frozen shoulder
		Primary Osteoporosis	Degenerative knee osteoarthritis	Degenerative Lumbar Spinal Stenosis	Fibromyalgia
		Spinal cord injury	Delayed-Onset Muscle Soreness	Functional ankle instability	Herniated lumbar disc-related sciatica
		hip fracture	Lumbar disease	lumbar intervertebral disc herniation	Lumbar Spinal Stenosis
		Myasthenia Gravis	Myelodysplastic Syndrome	Osteoarthritis of Knee	Osteoarthritis of the knee
		osteonecrosis of the femoral head	Osteopenia	Postpartum rectus abdominis separation	Rotator cuff injury
		Sarcopenia	shoulder periarthritis	Total knee arthroplasty	Active Knee Osteoarthritis
		Acute ankle injury	Acute knee synovitis	AI-Associatied Musculoskeletal Symptoms	cervical region and shoulder region myofascitis
		cervical spondylitis neck pain	cervical spondylopathy of vertebral artery	Cervical spondylosis of cervical type	Chronic ankle instability
		chronic ankle sprain	chronic rotator cuff injury	congenital muscular torticollis	Degenerative lumbar spondylolisthesis
		Degenerative osteoarthritis of the knee	Distal radial fracture	Early osteoarthritis of the knee	Early osteonecrosis of the femoral head with osteoporosis
		Elderly patients with hip fracture surgery	external humeral epicondylitis	Facial Synkinesis	failed back surgery syndrome

		Fibromyalgia syndrome	Functional Ankle Joint Instability	Hand Osteoarthritis	Hemifacial Spasm
		High level lumbar disc keyboard herniation	ICU acquired muscle weakness	Idiopathic Frozen Shoulders	Myofascial syndrome
		myofascitis	Neck function disorder	Nerve root and vertebral artery mixed cervical spondylopathy	Osteoarthritis of shoulder and knee
		Osteonecrisis of the femoral head	osteoporsis and osteopenia	osteoportic fracture	Patello-femoral pain syndrome
		periarthritis of shoulder (wind cold dampness)	Plantar Fasciitis	Primary Osteoporosis (Kidney-Yang Deficiency Type)	Primary type I osteoporosis
		reconstruction of anterior cruciate ligament	Rotator cuff injury	senile knee osteoarthritis	Senile osteoporosis
		Senile osteoporotic fracture	Shoulder hand syndrome (SHS) after stroke	Soft Tissue Injuries	Sphincter of Oddi Dysfunction
		Temporomandibular Disorders	terminal knee osteoarthritis	Third Lumbar Trans-verse Process Syndrome	Tic disorder
		Upper Crossed Syndrome	Weak muscle mass - Osteoporosis		
2	Neurological	Alzheimer's Disease	Parkinson's Disease	Restless Leg Syndrome	Facial nerve Palsy
		Stroke	Giddiness	Neurocognitive Disorders	Acute Ischemic Cerebrovascular Disease
		Vascular Dementia	Nerve Damage	Epilepsy	Cognitive Dysfunction

		Aphasia	Post-stroke Sequelae	Peripheral Nerve Injury	Peripheral Neuropathy
		Acute Ischemic Stroke	Hepatic Encephalopathy	Amyotrophic Lateral Sclerosis	Post-Stroke Fatigue
		Ischemic Stroke	Facial paralysis	Post-stroke insomnia	Multiple sclerosis
		Accupuncture	Vagus Nerve Stimulation	Postoperative Delirium	Trochlear Nerve Palsy
		Abducens Nerve Paralysis	Oculomotor Nerve Palsy	Cervical Radiculopathy	Precompetition Nervous Syndrome
		Spondylosis with radiculopathy	Cognitive Impairment	Bell's palsy	Upper Limb Spastic Paralysis after Stroke
		Poststroke Spasticity	Motor dysfunction after stroke	Sensorneural hearing loss	Delirium
		Urine retention due to spinal cord injury	Sequela of cerebral infarction leads to limb movement disorder	Autonomic nerve dysfunction during convalescence of stroke	Shoulder pain after stroke
		Perioperative symptoms of neurosurgery	Lower limb dysfunction during stroke recovery	Hand cramps after stroke	Balance Ability
		Repeated implantation failure	Dysphagia of pseudobulbar paralysis	Tracheotomy after stroke	Hemiplegia after cerebral infarction
3	Mental health	Anxiety Disorders	Opioid Withdrawal Syndrome	Post-traumatic Stress Disorder	Heroin Addiction
		Panic Disorder	Schizophrenia	Major Depressive Disorder	Suicidal Ideation
		Amphetamine Addiction	Insomnia	Addiction	Autism
		Drug Abuse	Bad State of Mind	Cigarette Addiction	Depression
		Alcohol Dependence	Geriatric Depression	Post-stroke Depression	Sleep Disorders

		Perioperative Anxiety	Bipolar Disorder	Substance Withdrawal Syndrome	Opioid-Related Disorders
		Postoperative Delirium	Permenopausal depressive disorder	Pathological Internet Use	Attention deficit hyperactivity disorder
		Depressive insomnia	Post-stroke Sleep Disorders	Shift work disorder	Climacteric depression
		Self perceptions of aging	Subthreshold Depression		
4	Cardiovascular and cerebrovascular	Heart Valve Disease	Stable Angina Pectoris	Acute Myocardial Infarction	Angina Pectoris
		Acute ST-segment Elevation Myocardial Infarction	Cardiovascular and Cerebrovascular Events	Percutaneous Transluminal Coronary Intervention	Arrhythmia
		Acute Coronary Syndrome	Venous Thromboembolism	Obstructive Hypertrophic Cardiomyopathy	Hypertension
		Cardiovascular Disease	Slow Arrhythmias	Lymphoedema	Atrial Fibrillation
		Varicose Veins	Atherosclerosis	Venous Ulcers of the Lower Extremities	Heart Failure
		Hypotension	Ischemic Cardiomyopathy	Congenital Heart Disease	Peripheral Artery Disease
		Myocardial Infarction	Elevated Blood Pressure	Coronary Heart Disease (CHD)	Intracerebral Hemorrhage
		Cerebrovascular Diseases	Non Obstructive Coronary Artery Disease	Atrial Premature Complexes	Chronic Coronary Syndrome

		Brain Ischemia	Coronary Artery Disease	Chronic Heart Failure	Cerebral Infarction
		Ischemic Heart Failure	Myocardial Ischemia (MSIMI)	Atherosclerotic Renal Artery Stenosis	Diastolic heart failure
		Unstable angina	Premature ventricular contractions	Chronic cardiac dysfunction complicated	Nonischemic Retinal Vein Occlusion(Syndrome of Static Blood Blocking Collaterals)
		Posterior circulation ischemia vertigo	Carotid atherosclerosis	Shoulder and Hand Syndrome after Cerebral Infarction	Dilated cardiomyopathy
		Lacunar infarction	Ventricular remodeling after acute myocardial infarction	Double heart disease	type 2 cardiorenal syndrome
		Angiocardiopathy	Ischemic cerebral infarction	Menopausal Vasomotor symptoms (VMS)	Spasm of the radial artery after coronary intervention
		Symptomatic Cerebral Artery Stenosis	Dyslipidemia	Cardiac Neurosis	
5	Digestive	Constipation	Fatty Liver	Cholecystitis	Irritable Bowel Syndrome
		Colonoscopy	Gallbladder Polyps	Gallbladder Surgery	Delayed Gastric Emptying Disorder
		Acute Pancreatitis	Crohn's Disease	Functional Indigestion	Gastrointestinal Surgery
		Gastroesophageal Reflux Disease	Diarrhea	Ulcerative Colitis	Non-atrophic Gastritis

		Gastrointestinal Disorders	Helicobacter Pylori Infection	Functional Dyspepsia	Postoperative Nausea and Vomiting
		Gastric ulcer	Functional Gastrointestinal Disorders	Choledocholithiasis	Non-erosive Reflux Disease
		Postoperative bowel obstruction	Gallstones	Chronic Atrophic Gastritis	Chemotherapy-induced Nausea and Vomiting
		Hepatic fibrosis	Paralytic ileus	Liver failure	Chronic Gastritis
		Nonalcoholic fatty liver disease	critical patients with enteral nutrition intolerance	Acute Drug-Induced Liver Injury by Anti-AIDS Drugs	Pediatric anorexia
		Ascites due to cirrhosis	Acute Gastrointestinal Injury	Acute radiation enteritis	Hemorrhoid
		Chronic Erosive Gastritis	Liver diseases	Portal hypertensive gastropathy in cirrhosis	Hepatolithiasis
		Biliary and pancreatic diseases	Primary biliary cholangitis	Hepatobiliary diseases	Alcoholic Liver Disease
		Biliary colic	Hepatic insufficiency	Cholelithiasis	Gastroesophageal reflux disease related Laryngologic reflux disease
		Postprandial Distress Syndrome	Critical Care Patients in ICU	Functional Intestinal Disorders	
6	Pain	Pain	Back Pain	Neck Pain	Chronic Pelvic Pain
		Myofascial Pain	Fibromyalgia	Bladder Pain Syndrome	Non-specific Low Back Pain
		Low Back Pain	Migraine Without Aur	Vestibular Migraine	Postherpetic Neuralgia

		Migraine	Chronic Tension-Type Headache	Sciatica	Rheumatic Pain
		Shoulder Pain	Cervicogenic headache	Headache	Breast pain
		Menstrual migraine	Pain of Mixture Indicator after Operation	Chest pain	Postpartum low back pain
		Musculoskeletal pain	Colonic spasm	Postoperative pain	Functional Anorectal Pain
		Knee pain	Pain caused by prostate cancer	Heel pain	
7	Respiratory	COVID-19	Chest and Lung Diseases	Cold and Flu	Community-acquired Pneumonia
		Idiopathic Pulmonary Fibrosis	Acute Pharyngitis	Acute Respiratory Failure	Upper Respiratory Tract Infection
		Tonsillitis	Interstitial Lung Disease	Chronic Obstructive Pulmonary Disease	Mechanical Ventilation
		Acute Lung Injury	Emphysema	VCI	Senile pneumonia
		Acute respiratory distress syndrome (ARDS)	chronic sinusitis	Respiratory diseases	Ground glass nodule of lung
		Respiratory Failure			
8	Gynecology	Infertility	Recurrent Miscarriage	Polycystic Ovary Syndrome	Laparoscopic Gynecological Surgery
		Uterine Adhesions	Acute Mastitis	Abnormal Uterine Bleeding	Menstrual Disease

		Early-onset Ovarian Insufficiency	Premature Ovarian Failure	Nausea and Vomiting in Pregnancy	Perimenopausal Hot Flashes
		Mastopathy	Pelvic Floor Organ Prolapse	postpartum urinary incontinence(PPUI)	Cesarean Delivery
		Menopausal Transition	Menopausal Syndrome	Endometriosis	Mastitis during Lactation
		Anesthesia for Caesarean Section	Non-lactating Mastitis	Menopausal Mood Disorders	Decreased Ovarian Function
		Pregnancy	Premature Miscarriage	Menstrual Pain	Failure After Assisted Reproductive Technology
		Pelvic Inflammatory Disease	Fibrocystic Disease of Breast	Atrophic Vaginitis	Threatened Miscarriage
		Abused Women	Perimenopause	Mammary gland hyperplasia	postoperative gastrointestinal disorder
		Adenomyosis	Uterine Fibroid Removal	Perimenopause syndrome	menopause related disease
		Chronic Pelvic Inflammation Disease	In Vitro Fertilization-Embryo Transfer (IVF-ET)	Adverse reactions after anesthesia	Ovarian hyperstimulation syndrome,OHSS
		Premenstrual syndrome, PMS	Chronic endometritis	Thin endometrium	Postpartum hypoglycemia
		Postpartum disease	Diminished ovarian reserve(DOR)	Breast carbuncle	poor ovarian response
		Luteal Phase Deficiency	Endometrial injury		
9	Tumour	Brain	Nasopharynx	Esophagus	Lung
		Rectum	Large Intestine	Cervix	Bile Duct
		Uterus	Lymph	Prostate	Blood

		Head and Neck	Stomach	Pancreas	Liver
		Bladder	Peritoneum	Oophoron	Breast
		Thyroid	Bone	Cancer-related Fatigue	Cancerous pain
		Gynecological tumors	Bronchogenic	Cancer Cachexia	Chemotherapy-induced alopecia
10	Urogenital	Overactive Bladder Syndrome	Erectile Dysfunction	Prostatic Hyperplasia	Hypermobility of the Bladder
		Stress Urinary Incontinence	Interstitial Cystitis	Prostatitis	Acute Kidney Injury
		Kidney Transplantation	Urinary Tract Stones	Urinary Tract Infections	Urinary Stones
		Renal Failure	Premature Ejaculation	Chronic Kidney Disease	Uremia
		End-stage Renal Disease	Hemodialysis	Acute Renal Colic	Neurogenic Bladder
		Mixed Urinary Incontinence	Chronic renai insufficiency	Renal Dialysis	Chronic Urinary Retention
		Urinary Incontinence	IgA Nephropathy at High Risk of Developing ESRD	Primary IgA Nephropathy	Glomerulonephritis
		Proteinuria	Idiopathic Membranous Nephropathy	Primary Refractory Nephritic Syndrome	Membranous nephropathy
		Urinary Retention	Polycystic Kidney disease CKD3 - 4	Male infertility	Acute pyelonephritis(APN)
		Urine leakage after indwelling catheter	Reproductive medical	Nocturia	Oligospermia and asthenospermia
		Renal calculi	Idiopathic asthenospermia	Lupus nephritis	Renal colic

11	Eyes, Nose, Ear and Dental	Tinnitus	Allergic rhinitis	Deafness	Mucositis of the Oral Cavity
		Dry Eye	Vitreoretinal Diseases	Chronic Subjective Tinnitus	Periodontitis
		Chronic Hypertrophic Rhinitis	Sudden Deafness	Oral Erosive Lichen Planus	Glaucoma
		Retinal Vein Obstruction	Olfactory Disorder	Sore throat	Xerostomia of hemodialysis patients
		Dysphonia	Vocal cord nodules	Oral leukoplakia	Dysphagia
		Thyroid associated ophthalmopathy	vocal cord leukoplakia	Visual display terminal syndrome	Chronic sinusitis
		Recurrent aphthous stomatitis	Temporomandibular Joint Disorder	Burning mouth syndrome	
12	Metabolic and endocrine	Obesity	Thyroidectomy	Goiter	Diabetic Nephropathy
		Diabetes and all its Complications	Gestational Diabetes	Hashimoto's Thyroiditis	Diabetes Mellitus
		Gout	Diabetes	Metabolic Syndrome	Hyperlipidemias
		Clinical Anesthesia	Hyperuricemia	MHD population mineral bone metabolism disorder	Impaired Glucose Regulation
		Low immunity	Decrease in immune function	Thyroid nodules	Gouty Arthritis
		abnormal glucose tolerance	Meibomian gland dysfunction(MGD)	endocrine therapy-related hot flashes	
13	Skin	Eczema	Melasma	Vitiligo	Scarring
		Acne	Atopic Dermatitis	Seborrheic Alopecia	Herpes Zoster

		Psoriasis	Psoriasis	Urticaria	Acute radiodermatitis
		Rosacea	Prurigo nodularis	Hand-foot skin reaction	Uremic pruritus
14	Rheumatic diseases	Rheumatoid Arthritis	Ankylosing Spondylitis	Systemic Lupus Erythematosus	Sjogren's Syndrome
		Chronic Rheumatic Diseases	Rheumatism	polymyalgia rheumatic,PMR	
15	Infection and parasite	AIDS	Herpes	Tuberculosis	Hepatitis C
		Hepatitis B	Sepsis	Viral hemorrhagic fevers,VHFs	Nonalcoholic steatohepatitis
		HPV infection	Influenza A	Postoperative shiver	
16	TCM	Qi arrest and blood stasis syndrome	Qi deficiency syndrome	Kidney Yin deficiency syndrome	Kidney-essence deficiency patients, premature aging population
		Liver depression and spleen deficiency type sub-health	Cervical spondylosis of Yang deficiency type	Tinnitus of damage of kidney-essnce	Damp heat in the spleen and the stomach
		Spleen-qi deficiency syndrome	Liver Depression and Fire-type Insomnia	Depression with kidney deficiency	
17	Hematological	Myelodysplastic Syndromes	Primary Thrombocythemia	Thrombocytopenia	Hemophilia A
		Idiopathic Thrombocytopenic Purpura	Hematopoietic Stem Cell Transplantation	Severe Aplastic Anemia	Iron-Deficiency Anemia
		Postpartum Anemia	Chronic myeloid leukemia(CML)	Leukopenia	Postoperative hypercoagulability status
		Acute myeloid leukemia	Blood deficiency syndrome		

18	Other	Chronic Fatigue Syndrome	Healthy	Acute Mountain Sickness	elderly fall
		Acquired weakness in mechanically ventilated ICU	college students with disabilities	depth of anesthesia	Geriatric disease
		Fatigue	Frailty syndrome	Functional reorganization	medium and large size abdominal surgery
		Geriatrics	high-altitude de-acclimatization syndrome	hospitalized elderly patients near the end of life	Premature Hair Graying
		not disease-related	Physiological effects induced by microgravity	pre-frail	Selective surgery
		refractory wound	Retinal oxygen saturation in healthy subjects	Sarcopenia	Suboptimal Health Status
		Senile Diseases	shelter syndrome	Sub-health state	Wilson's disease
		Swimming-specific athletic performance is limited	The aging of normal people	Fatigue state	

e Table 2 PRO Tests Used Most Frequently

	**Test**	**Frequency of Use, No. (%)**
1	VAS	463 (25.7)
2	TCMSS	264 (14.6)
3	SF-36	213 (11.8)
4	PSQI	200 (11.1)
5	SAS	170 (9.4)
6	SDS	161 (8.9)
7	HAMD	119 (6.6)
8	HAMA	117 (6.5)
9	WOMAC	110 (6.1)
10	MMSE	78 (4.3)
11	MoCA	71 (3.9)
12	TCM Symptom Rating Scale	69 (3.8)
13	SAQ	53 (2.9)
14	NRS	53 (2.9)
15	ODI	50 (2.8)

VAS, visual analogue scale; TCMSS, TCM symptom score; SF-36, Short-Form 36-item Health Survey; PSQI, Pittsburgh Sleep Quality Index; SAS, Self-Rating Anxiety Scale; SDS, Self-Rating Depression Scale; HAMD, Hamilton Depression Scale; HAMA, Hamilton Anxiety Scale; WOMAC, The Western Ontario and McMaster Universities Osteoarthritis Index; MMSE, Mini-mental state examination; MoCA, Montreal Cognitive Assessment; TCM Symptom Rating Scale; SAQ, Seattle Angina Questionnaire; NRS, Numeric rating scale; ODI, Oswestry Disability Index.
